# Future leader to watch – Aaron Savage

**DOI:** 10.1242/bio.059068

**Published:** 2021-10-20

## Abstract

First Person is a series of interviews with the first authors of a selection of papers published in Biology Open, helping early-career researchers promote themselves alongside their papers. Aaron Savage is first author on ‘ Germline competent mesoderm: the substrate for vertebrate germline and somatic stem cells?’, published in BiO. Aaron is a postdoctoral research associate at the Biodiscovery Institute, University of Nottingham, United Kingdom, investigating how stem cells can be used in regenerative medicine and how we can understand stem cell biology using embryonic and post-embryonic development.


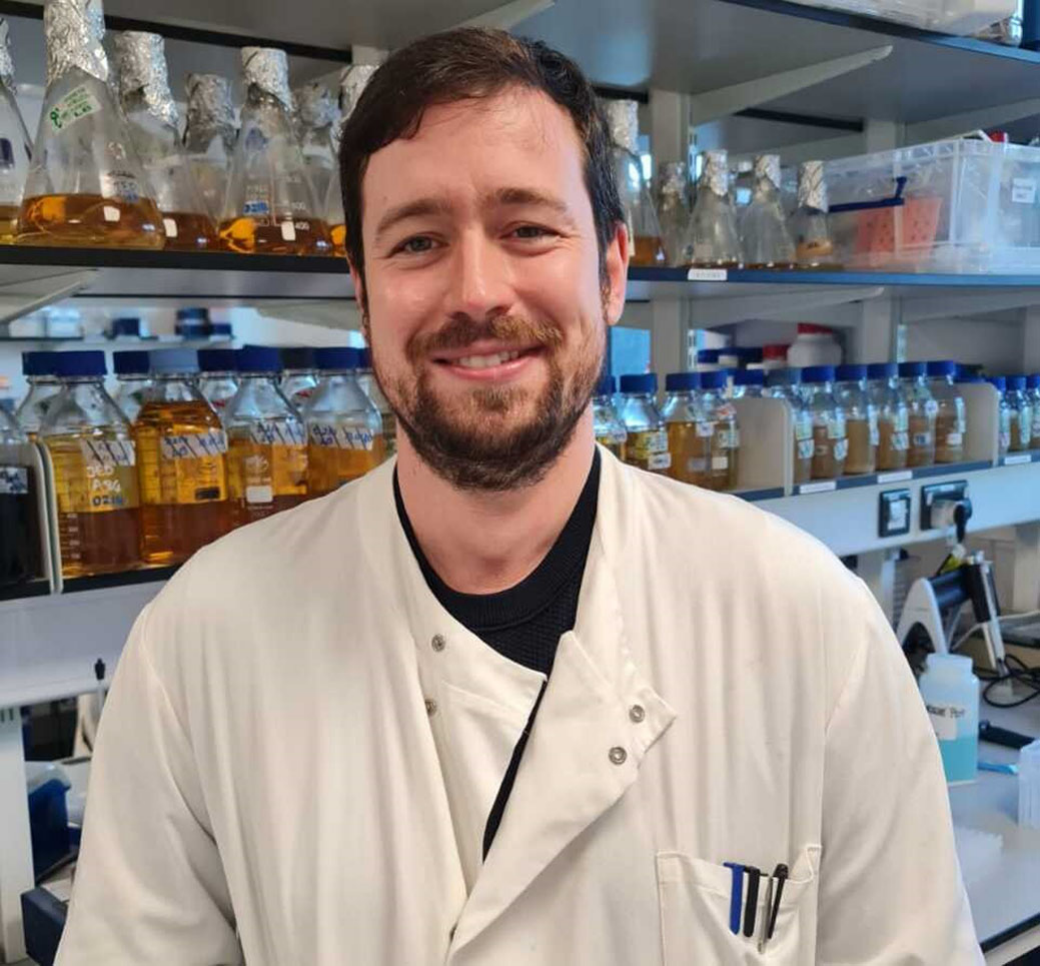


**Aaron Savage**

**What is your scientific background and the story of how you got to where you are today?**

I have always been interested in evolution from a young age, stemming from a fascination with dinosaurs, which many children experience. I was always interested in a career in science. I studied Biochemistry and Genetics at the University of Nottingham and found the links between developmental biology and evolution fascinating. This was compounded during my undergraduate project working with Dr Martin Gering, where I worked on the transcriptional regulation of haematopoietic stem cells (HSCs), using zebrafish. Seeing HSCs in circulation in a living embryo for the first time was something I'll never forget. I subsequently continued the project at the master's level, generating and analysing new transgenic zebrafish lines.

I then began a PhD at the University of Sheffield, under the supervision of Prof. Tim Chico and Dr Rob Wilkinson, where I studied how blood vessel development is regulated by calcium signalling, again in zebrafish. I developed CRISPR interference technology to analyse gene function, and how this impacts on cell signalling in a cell-specific manner. During my PhD I was fortunate to be mentored by supervisors who encouraged me to take the project in my own direction, which led to my first author publication.

During this time, I was fortunate enough to meet AJ, and later Ramiro, the co-authors of the Review. Working together with them has been a great experience and helped me expand how I view evolution and development, and their encouragement is the reason this Review exists.

I am currently working on developing *in vitro* CRISPR knock-in for gene delivery in lung cells. This has been an exciting part of my career in which I developed many new skills.

**What is the most important take-home message of your Review?**

The Review focusses on the idea that cell types that may not appear to be related display common features, which might suggest they are related. In this case, I focus on HSCs and primordial germ cells (PGCs), the cells that form sperm and eggs.

In many model organisms, such as zebrafish, PGCs are separated from the rest of the cells of the body in early development, but this is unlikely to be the case in humans, which may separate PGCs at a later developmental stage, similar to findings in other mammals. Interestingly, studies have shown a close association of expression profile between PGCs and HSCs. We suggest that they may be related or share a common ancestor during development in many species, including humans.

“I was surprised about the number of studies that show similarities between HSCs and PGCs, even during human development, which did not comment on the possibility of a developmental link.”

**What has surprised you the most while researching this Review?**

I was surprised about the number of studies that show similarities between HSCs and PGCs, even during human development, which did not comment on the possibility of a developmental link. What was very surprising was evidence for cells with overlapping expression profiles, migrating simultaneously to regions within human embryos associated with both blood and germline, which is an incredibly strong suggestion that the cell types are linked.

Another surprising finding was the number of articles that show a capacity for stem cells of one type to produce seemingly unrelated cells. An example of this is HSCs being induced to produce neural cells. This indicates a plasticity between cell types that might have important impacts on regenerative medicine.

**What do you feel is the most important question that needs to be answered to move the field forward?**

The most important thing to definitively clarify is how specific stem cell types (for example HSCs) might be related to the germline, within species that do not use germ plasm to specify germline. There have been many studies that indicate that expression profiles may overlap, but none have tested whether stem cells and germline have a shared origin.

This understanding not only improves our view of how we (and other organisms) evolved but has implications for the future of regenerative medicine – understanding how stem cells form in during human development might improve the potential to generate stem cells and their descendants *in vitro*, allowing for better future therapies.

**What changes do you think could improve the professional lives of early-career researchers (ECRs)?**

I think an overhaul of how ECRs can develop independence is needed. A lot of ECRs have forward-thinking ideas that often go unfunded because either the idea, or funding someone with a limited track record, could be considered risky, or both. The recent UKRI initiative of having multiple PIs on a grant is a step in the right direction, which enables ECRs to show that they were involved with the formulation of the hypothesis. The possibility of a wider range funding opportunities in this context could significantly improve ECR life – who doesn't want to work on an idea they were directly involved in developing?

What is important is that the ideas of ECRs can be heard and opportunities like the Biology Open Future Leader Reviews are an excellent way for young researchers to show that their ideas have potential.

“A lot of ECRs have forward-thinking ideas that often go unfunded because either the idea, or funding someone with a limited track record, could be considered risky, or both.”

**What's next for you?**

From January, I will be working in Jessica Whited's lab at Harvard University, which is a really exciting prospect! I will be working on how blood vessels interact with other cell types during the limb regeneration process in axolotls.
